# Dog ownership and healthy brain aging: a pilot study

**DOI:** 10.7717/peerj.21560

**Published:** 2026-07-31

**Authors:** Katie Potter, Douglas N. Martini, Colleen J. Sands, Ryan S. Falck, Rajakumar Nagarajan

**Affiliations:** 1 Department of Kinesiology, University of Massachusetts at Amherst, Amherst, MA, United States of America; 2 School of Biomedical Engineering, The University of British Columbia, Vancouver, British Columbia, Canada; 3 Human Magnetic Resonance Center, Institute for Applied Life Sciences, University of Massachusetts at Amherst, Amherst, MA, United States of America

**Keywords:** Aging, Older adult, Human-animal bond, Dog ownership, Cognitive aging, Brain aging, Physical activity, Social connectedness, Dog walking, Dementia prevention

## Abstract

**Background:**

Dog ownership may benefit brain and cognitive health by supporting physical activity and social connectedness, among other mechanisms. Studies that include brain health outcomes (*e.g.*, neuroimaging outcomes) are lacking. As the population ages, studies investigating whether and how dogs support healthy brain aging are needed, as dog ownership and dog walking are modifiable targets for dementia prevention efforts. This study piloted all methods for a full-scale investigation of how dog ownership in older adulthood impacts brain aging.

**Methods:**

This was a cross-sectional study. Participants (*n* = 25; *n* = 13 dog owners) completed physical activity monitoring (activPAL + diary for 7 days) and questionnaires relating to social connectedness. They also completed cognitive testing (NIH Toolbox Cognition Battery) and brain magnetic resonance imaging (MRI). Brain structural volumes (*e.g.*, total gray matter volume) and cortical thickness were quantified. Process outcomes of interest included sample characteristics and data completeness. Descriptive statistics were used to summarize the data overall and by group.

**Results:**

Participants were 74.8 ± 3.9 years old, 60% were female, 100% were non-Hispanic White, 84% had at least a 4-year college degree, and 76% reported very good or excellent health. Most (84%) provided complete survey, physical activity, cognition, and brain MRI data. Dog owners took 8,972 ± 5,650 mean steps per day, while non-dog owners took 7,107 ± 2,989 mean steps per day. Most dog owners (11/13; 85%) reported dog walking, and most (11/13; 85%) reported meeting people in their neighborhood through their dog. Dog owners scored 6.8 ± 2.9 on the PROMIS Social Isolation v2.0–Short Form 4a (scale 4–20, higher scores = more isolation), while non-dog owners scored 6.7 ± 2.7. Dog owners scored 112.2 ± 12.8 on the NIH Fluid Cognition Composite Score (100 indicates average function), while non-dog owners scored 116.3 ± 10.3. Total gray matter volume was 599,156 ± 10,035 mm^3^ for dog owners and 588,028 ± 12,945 mm3 for non-dog owners. Total cortical thickness was 2.35 ± 0.03 mm^2^ (right hemisphere) and 2.35 ± 0.03 mm^2^ (left hemisphere) for dog owners and 2.30 ± 0.04 mm^2^ (right hemisphere) and 2.32 ± 0.04 mm^2^ (left hemisphere) for non-dog owners.

**Conclusions:**

This pilot research was performed to inform a larger study on the brain and cognitive health impact of dog ownership in older adulthood. This study confirmed feasibility of data collection procedures while identifying important modifications needed for the success and rigor of the hypothesis-testing study, namely that recruitment strategies must be modified in the larger study to ensure a more representative study sample with greater variability in mechanisms of interest (*e.g.*, social connection, physical activity, dog walking).

## Introduction

By 2060, nearly one in four Americans will be over the age of 65 (Vespa, Medina & Armstrong, 2020). Although American society should benefit in many ways from having more emotionally stable (Carstensen, 2021), prosocial residents (Ep et al., 2021), there are also societal challenges associated with the nation’s advancing age. One challenge is the projected increase in rates of Alzheimer’s dementia. In 2024, an estimated 6.9 million Americans were living with Alzheimer’s dementia (Alzheimer’s Association, 2024). By 2060, that number is projected to double to 13.8 million (Alzheimer’s Association, 2024). Although the top risk factor for dementia is not modifiable (age) (Alzheimer’s Association, 2024)), an estimated 45% of dementia cases worldwide can be attributed to modifiable risk factors (Livingston et al., 2024). Physical activity is one factor consistently associated with decreased risk of all-cause, Alzheimer’s, and vascular dementia (Iso-Markku et al., 2022), but most older Americans (60%) are not physically active at recommended levels (Whitfield, Hyde & Carlson, 2021). Social disconnection is another modifiable risk factor for dementia (Evans et al., 2019; Kuiper et al., 2015; Livingston et al., 2020; Penninkilampi et al., 2018) that needs critical attention in the US. Older adults are more likely to experience certain risk factors for social isolation and loneliness, including death of loved ones, worsening health, and retirement (National Academies of Sciences, 2020). About 25% of older Americans are considered socially isolated (Cudjoe et al., 2020) and 20% report frequent loneliness (another 30% report occasional loneliness) (Hawkley, Kozloski & Wong, 2017).

**Figure 1:**
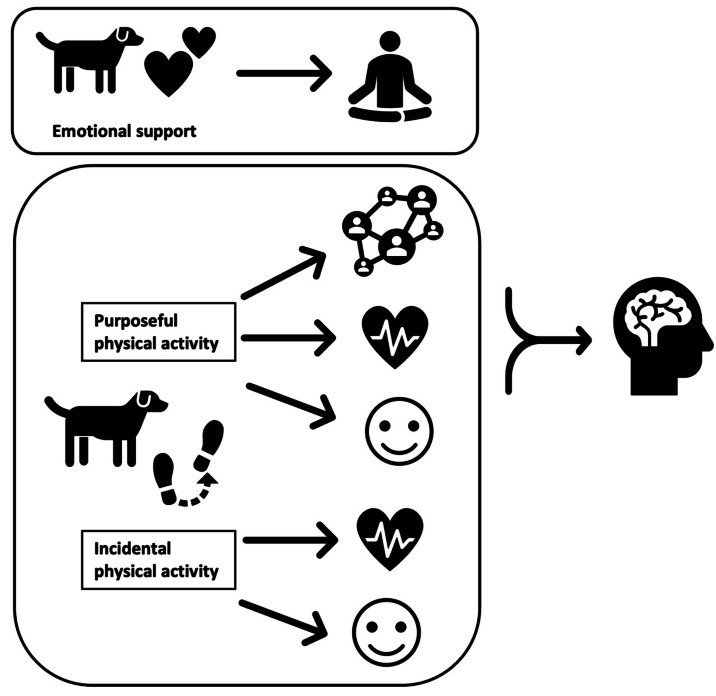
Plausible pathways linking dog ownership with healthy brain aging. Emotional support from dog companions may protect the brain by buffering stress. Purposeful and incidental physical activity may protect the brain by supporting cardiometabolic health and preventing depression. Purposeful physical activity *via* dog walking may also facilitate social interaction.

Pet ownership in older adulthood, particularly dog ownership, may promote both physical activity and social connectedness (Enders-Slegers & Hediger, 2019; Gee & Mueller, 2019) (see Fig. 1). The rationale is that taking care of a dog can lead to incidental and purposeful physical activity throughout the day (Peacock et al., 2020), and that dogs facilitate social interactions by increasing perceptions of friendliness and serving as conversation starters or ‘ice breakers’ (McNicholas & Collis, 2000; Wells, 2004). Indeed, a growing body of literature demonstrates that older adult dog owners are more physically active than non-dog owners (Curl, Bibbo & Johnson, 2017; Dall et al., 2017; Feng et al., 2014; Hoerster et al., 2011; Knight & Edwards, 2008; Mein & Grant, 2018; Mueller et al., 2021; Shibata et al., 2012; Thorpe et al., 2006; Toohey et al., 2013) ^,^ (Gretebeck et al., 2013) and the study of pets, particularly pet dogs, as social facilitators has a 40+ year history (Bulsara et al., 2007; McNicholas & Collis, 2000; Messent, 1983; Mugford & M’Comisky, 1975; Taniguchi et al., 2018; Wood et al., 2017; Wood et al., 2015; Wood, Giles-Corti & Bulsara, 2005). In 1983, a pioneering study by Messent (1983) found that dog owners walking in a park experienced more chance conversations with other park users when walking with their dog than when walking the same route without their dog. The ability of dogs to catalyze casual social interactions has since been replicated in other studies (McNicholas & Collis, 2000; Rogers, Hart & Boltz, 1993), and work by Wood et al. (2015) has demonstrated that pet-facilitated social encounters sometimes evolve into friendships and sources of social support (Wood et al., 2015). These benefits are more likely to be experienced by dog owners who walk their dog, however, even those who do not walk their dog may receive social connection benefits from dog ownership (directly from the dog-owner relationship).

Dog-facilitated physical activity and social connectedness may protect the aging brain *via* various pathways. Physical activity directly reduces the hallmarks of brain aging (*e.g.*, dysregulated energy metabolism, neuronal damage from oxidative stress) (Wahl, Cavalier & LaRocca, 2021) and the physically active or fit brain has greater total volume, regional gray matter volumes (*e.g.*, hippocampal volume (Erickson et al., 2009; Gu et al., 2020; Hamer, Sharma & Batty, 2018; Tan et al., 2017; Varma et al., 2015)) and white matter volume (Sexton et al., 2016). Higher physical activity or fitness has also been associated with less cortical thinning (Falck et al., 2020; Williams et al., 2017) and greater functional connectivity within resting state brain networks most sensitive to aging (Salami, Pudas & Nyberg, 2014) and cognitive decline (Buckley et al., 2017). The brain impacts of social connectedness are less established. Informal social interactions experienced by dog walkers may support *social bridging*, which occurs when people have frequent interactions with casual friends, neighbors, or other ‘weak ties’ (Manchella et al., 2024; Perry et al., 2022). Alternatively, a close human-dog relationship may support *social bonding,* which results from regular interaction with close friends and family members. Social bridging is believed to build cognitive reserve (Manchella et al., 2024; Perry et al., 2022), whereas bonding may protect the brain *via* stress buffering (Manchella et al., 2024; Perry et al., 2022). A recent study with cognitively impaired and unimpaired older adults found social bridging associated with greater gray matter density in limbic system structures (sensory processing regions) and social bonding associated with greater gray matter density in frontal lobe regions (stress modulation regions), reflecting the mechanistic pathways (Manchella et al., 2024). The brain benefits of physical activity and social connectedness translate to improved cognition and/or a reduced risk of cognitive decline in older adults, particularly in the domains of executive function and episodic memory (Erickson et al., 2019; Iso-Markku et al., 2024; Samtani et al., 2022).

Given the links between dog ownership and these behavioral risk factors for dementia, researchers have begun exploring the brain and cognitive health benefits of dog ownership in older adulthood (Applebaum et al., 2022; Batty et al., 2017; Branson et al., 2016; Branson & Cron, 2022; Friedmann et al., 2023; Friedmann et al., 2020; Li et al., 2023; Taniguchi et al., 2023). The existing literature is limited in key ways. For example, many studies examine the cognitive benefits of pets, not dog specifically, which may weaken associations if cats and other common pets do not impact physical activity and social connectedness to the extent dogs do. Further, crude assessments of dog ownership status (yes/no) and participation in dog walking or general pet caretaking (*e.g.*, >0 hrs per week *vs* 0 hrs per week) are common and do not provide the level of detail needed to understand how, for whom, and under what conditions dogs may positively impact brain and cognitive health. Finally, only one study has looked at the impact of dog ownership on brain-level indicators of cognitive aging using neuroimaging methods(McDonough et al., 2022). Studies that collect cognitive data paired with behavioral mechanism (*e.g.*, physical activity, social connection) and brain-level mechanism data are needed to clarify whether and how dogs impact dementia risk and move the field closer to intervention development studies and thereby clinical and public health impact.

Pilot studies are a critical first step in research. They determine feasibility and identify modifications needed in the hypothesis-testing study (Leon, Davis & Kraemer, 2011), which helps ensure efficient use of time and resources. The purpose of the current study was to pilot procedures, including physical activity, social connectedness, cognitive function, and brain imaging data collection procedures, for a larger investigation of the brain health benefits of dog ownership in older adulthood. The goal is for this work to inform an efficient, full-scale study that will explain *how* dogs impact brain and cognitive aging and *who* (*e.g.*, adults who live alone) benefits most from dog companionship in older adulthood. This will allow for the development of evidence-based programs and policies that leverage the human-dog bond to prevent or delay dementia and that are tailored for those who need them most.

## Materials & Methods

### Study design and population

This was a cross-sectional study. Community-dwelling older adults ages 70–84 years who were retired or working ≤20 hours/week, free from neurological disease, and able to speak and read English were eligible to participate. Dog owning participants had to be the main/joint caretaker of their dog and their dog needed to be in good health. Ineligibility criteria included a current diagnosis of major depression, history of major psychiatric illness (not including general anxiety disorder or depression), neurological condition (multiple sclerosis (MS), Parkinson’s, Dementia, Mild Cognitive Impairment (MCI)) or brain injury (traumatic or stroke), current alcohol or substance abuse, current treatment for cancer (except non-melanoma skin), inability to walk unaided for at least 2 min, recent hospitalization for a condition affecting mobility, or a positive test for COVID-19. Individuals who could not undergo MRI (*e.g.*, due to a pacemaker or other non-MRI safe implant) were allowed to participant in all study procedures except the MRI. All participants provided written informed consent. This study was approved by the University of Massachusetts Amherst Institutional Review Board (protocol ID: 2710). Data were collected in 2022.

### Procedures

Participants were recruited through university recruitment databases and email newsletters, as well as flyers posted in the local area. Prospective participants also learned about the study from a university press release and local tv feature on the study. Participants completed informed consent *via* Zoom before attending two visits to campus. During the first visit, they completed a cognitive screener (the Mini-Cog (Borson et al., 2003)) and a battery of cognitive tests, and were issued a physical activity monitor and log booklet. They were instructed to wear the monitor and track leisure-time physical activity for the next 7 days, and to complete one set of online questionnaires during this time frame. After completing the physical activity monitoring protocol, participants returned to the University for their second study visit, which involved an MRI brain scan. Participants received $20 for completing the first visit, $20 for completing physical activity monitoring, and $20 for the MRI scan (up to $60, total).

### Measures

#### Physical activity & dog walking

activPAL3 monitors (PAL Technologies, Glasgow, Scotland) were used to assess physical activity. The activPAL is a small (23.5 × 43 × 5 mm), lightweight (9 grams), thigh-worn device that uses accelerometer-derived information to classify standing, walking, and sitting/lying behaviors. It is commonly used in research with community-dwelling older adults (Blackwood et al., 2022) and has been shown to measure step number and cadence in this population with an absolute percentage error of <1% (Grant et al., 2008). Participants were asked to wear the activPAL 24 hours/day (except while swimming) for 7 consecutive days. activPAL data were processed using PAL software. To be included in analyses, participants needed to wear the device for at least 23 hours on at least 4 days, including 1 weekend day. Participants were also asked to log all leisure-time physical activity, including any dog walking, for the same 7 days they wore the activPAL monitor.

#### Social connectedness

The PROMIS Social Isolation v2.0–Short Form 4a (Hahn et al., 2014) was used to assess perceived social isolation. Items include ‘I feel left out’, ‘I feel that people barely know me’, ‘I feel isolated from others’, and ‘I feel that people are around me but not with me’. Response options are on a 5- point scale (never-1, rarely-2, sometimes-3, usually-4, always-5). Participants also completed a set of questions from a previous study investigating pets as social facilitators (Wood et al., 2015). The questions ask about getting to know people in the neighborhood generally, as well as through pets specifically, and whether any pet-facilitated contacts have developed into friends or sources of social support (emotional, informational, appraisal, or instrumental support) (Wood et al., 2015).

#### Cognitive function

The iPad-administered NIH Toolbox Cognition Battery for ages 8–85 years was used to assess cognitive function (Gershon et al., 2010). The Fluid Cognition Composite Score was used as the primary cognitive outcome. Fluid abilities are used for problem solving, thinking and acting quickly, encoding new episodic memories, and adapting to novel situations (Slotkin et al., 2021), and are susceptible to decline with aging (Slotkin et al., 2021). The Fluid Cognition Composite Score is produced automatically by the NIH Toolbox software and is derived by averaging the normalized scores of five fluid ability measures, then deriving scale scores based on this new distribution (Slotkin et al., 2021). The measures include tests of attention (Flanker Inhibitory Control and Attention Test), episodic memory (Picture Sequence Memory Test), working memory (Sorting Working Memory Test), executive function (Flanker plus Card Sort Test) and processing speed (Pattern Comparison Processing Speed Test). The Fluid Cognition Composite Score has shown acceptable to excellent test-retest reliability and strong convergent and discriminant validity when tested against gold-standard neuropsychological tests in healthy adult and older adult samples (Heaton et al., 2014; Scott, Sorrell & Benitez, 2019). ‘Age corrected’ standard scores, provided in the NIH Toolbox Scoring & Interpretation Guide, are reported. Scores around 100 indicate average ability compared to others nationally. Age-adjusted national percentiles (also provided by the NIH Toolbox) are also reported for context.

#### Brain health

Brain structural metrics, including cortical thickness and cortical and sub-cortical brain volumes (Aljondi et al., 2019; Dickerson, Wolk & Alzheimer’s Disease Neuroimaging Initiative, 2012; Habeck et al., 2020; Mungas et al., 2005; Pacheco et al., 2015), were used as biomarkers of brain health in this study. Imaging was conducted on a 3T MAGNETOM Skyra scanner (Siemens Healthineers) using a 32-channel head coil. High-resolution three-dimensional isotropic T1-weighted images were acquired with a repetition time (TR) of 2400 ms, an echo time (TE) of 3.36 ms, a field of view (FOV) of 256 mm, and an isotropic voxel size of one mm^3^. FreeSurfer version 6.0 image analysis suite (Martinos Centre for Biomedical Imaging, Laboratory for Computational Neuroimaging; http://surfer.nmr.mgh.harvard.edu/) was used to calculate brain volume and cortical thickness. FreeSurfer consists of two processing streams, a surface-based stream and a volume-based stream. Data processing included skull-stripping (Ségonne et al., 2004), motion correction (Reuter, Rosas & Fischl, 2010), Talairach transformation (Fischl et al., 2004), and atlas registration (Fischl et al., 1999). Output was visually inspected following surface reconstruction and segmentation to ensure quality and accuracy of the data. Manual correction resolved inaccuracies due to the automated skull stripping procedure, incorrect white matter segmentation, automated topological fixer inaccuracies, and imprecise pial surface estimation. White matter control points were added in the event of intensity normalization failures. Within FreeSurfer, the recon-all -qcache command was used to resample the data onto the cohort mean subject and smooth the images with a 10-mm full width at half-maximum (FWHM) Gaussian Kernel. Brain structural volumes and cortical thickness were then extracted for the following global measures and regions of interest: (1) total gray matter volume (mm^3^); (2) total white matter volume (mm^3^); (3) right and left hippocampal volumes (mm^3^); and (4) total right and left hemisphere cortical thickness (mm^2^).

### Statistical analyses

Descriptive statistics were used to summarize process variables of interest (sample characteristics, data completeness), mechanistic variables of interest (social connectedness, physical activity, dog walking), and outcome variables of interest (cognitive function, brain health (volume, cortical thickness)). We limited analyses to summary statistics, as it is not appropriate to use small pilot studies to estimate group differences or generate effect sizes (Kraemer et al., 2006; Leon, Davis & Kraemer, 2011; Teresi et al., 2022).

## Results

Forty-nine individuals completed the study screening questionnaire. Twenty-six individuals completed informed consent. Twenty-five individuals completed the study (one individual dropped out after completing informed consent). Participants (13 dog owners, 12 non-dog owners) were 74.8 ± 3.9 years of age, on average, 60% were female, 100% were non-Hispanic White, 84% had at least a 4-year college degree, and 76% reported very good or excellent health. See Table 1 for complete study demographics overall and by group. Most participants (21 of 25; 84%) provided complete, useable data, including brain imaging data. Two participants did not complete the MRI portion of the study due to non-MRI safe implants. Of the 23 participants who completed MRI scanning, 91% (21 of 23) provided complete, useable data. One participant did not return their physical activity log booklet and one participant’s MRI data were unusable due to excessive motion during scanning.

**Table 1:** Study demographics.

	**Overall (*n* = 25)**	**Dog-owner (*n* = 13)**	**Non-dog-owner (*n* = 12)**
Sex			
No. (%) female	15 (60%)	7 (54%)	8 (67%)
Age			
Mean (SD) years	74.8 (3.9)	75.2 (4.6)	74.5 (3.2)
Education Level			
High school or 2-year college	4 (16%)	4 (31%)	0 (0%)
4-year college	7 (28%)	4 (31%)	3 (25%)
Graduate or professional degree	14 (56%)	5 (38%)	9 (75%)
Race/ethnicity			
No. (%) non-Hispanic white	25 (100%)	13 (100%)	12 (100%)
Household Income^a^			
<$100,000	12 (57%)	8 (80%)	4 (36%)
≥$100,000	9 (43%)	2 (20%)	7 (64%)
Marital Status			
No. (%) Married	16 (64%)	8 (62%)	8 (67%)
Household Status			
No. (%) Living alone	16 (64%)	8 (62%)	8 (67%)
Community setting			
Suburban or urban	17 (68%)	8 (62%)	9 (75%)
Rural	8 (32%)	5 (38%)	3 (25%)
Health status			
Excellent or very good	19 (76%)	7 (54%)	12 (100%)
Good	6 (24%)	6 (46%)	0 (0%)

**Notes.**

a 4 participants preferred not to answer (3 dog owners).

### Physical activity and dog walking

Participants averaged 8,076  ±  4,578 steps per day. See Table 2 for daily step data by group. Most dog owners (11/13; 85%) reported at least one dog walk during the 7 days they tracked their physical activity. Frequency and total volume of dog walking was highly variable, ranging from 0–19 walks over the 7 days (average = 7.9  ±  6.6 walks) and from 0-1250 total minutes of dog walking (average = 387.3  ±  361.4 minutes).

**Table 2:** Physical activity, social isolation, and cognitive function by group.

	Overall (*n* = 25)	Dog owner (*n* = 13)	Non-dog owner (*n* = 12)	Mean difference
	mean ± SD	mean ± SD	mean ± SD	
Daily steps	8,076 ± 4,578	8,972 ± 5,650	7,107 ± 2,989	1,865.4
Social isolation^a^	6.7 ± 2.8	6.8 ± 2.9	6.7 ± 2.7	0.1
Cognitive function^b^	114.1 ± 11.6	112.2 ± 12.8	116.3 ± 10.3	−4.1

**Notes.**

a PROMIS social isolation v2.0–short form 4a, scores range from 4-20, higher score indicates greater isolation.

b Age adjusted standard scores for NIH Toolbox Fluid Cognition Composite Score; scored around 100 indicate average fluid cognitive ability compared to others nationally.

### Social connection

Participants had low levels of social isolation. The raw scores on the PROMIS Social Isolation measure (Hahn et al., 2014), which can range from 4-20 with lower scores indicating less isolation, averaged 6.7 ± 2.8. See Table 2 for social isolation data by group. Most participants (23/25; 92%) reported having met people in their neighborhood. Most dog owners reported meeting people in their neighborhood through their dog (11/13; 85%) and that someone they met through their dog had become a friend and/or provided social support.

### Cognition

Participants scored 114.1 ± 11.6, on average, on the NIH Toolbox Fluid Cognition Composite Score (100 indicates average function). See Table 2 for cognition data by group. Almost half the sample (11/25; 44%) performed above the 80th percentile based on age-adjusted, national percentile rankings provided in the NIH Toolbox Scoring & Interpretation Guide, including seven participants (28% of sample) who scored at or above the 96th percentile.

### Brain health

Brain volume and cortical thickness data by group are reported in Table 3. Data processing factored in age, sex, education, and total intracranial volume.

**Table 3:** Marginal mean brain volume and cortical thickness in dog owners and non-dog owners ^a^.

	**Dog owners (*n* = 10)**	**Non-dog owners (*n* = 12)**	**Contrast**
Total gray matter volume (mm^3^)	599,156 (10,035)	588,028 (12,945)	11,128 (16,525)
Total white matter volume (mm^3^)	473,296 (15,390)	456,654 (19,853)	16,642 (25,343)
Hippocampal volume (mm^3^)–R hemisphere	3,976 (113)	3,921 (145)	55 (186)
Hippocampal volume (mm^3^)–L hemisphere	3,864 (92.5)	3,788 (119.3)	75.7 (152)
Total cortical thickness (mm^2^)–R hemisphere	2.35 (0.03)	2.30 (0.04)	0.06 (0.05)
Total cortical thickness (mm^2^)–L hemisphere	2.35 (0.03)	2.32 (0.04)	0.03 (0.05)

**Notes.**

a Analyses controlled for age, sex, education, total intracranial volume

## Discussion

The purpose of the current study was to pilot procedures for a larger investigation of how dog ownership in older adulthood impacts brain and cognitive aging. The main finding is that the study design and data collection methods appear feasible, but recruitment methods must be modified to ensure a more diverse and representative sample in the hypothesis-testing study. In this small convenience sample, there were low levels of social isolation and high levels of neighborhood social connectedness. Physical activity and dog walking behavior varied greatly, but, on average, levels were high. Fluid cognitive ability was above average, with many participants demonstrating exceptional cognitive ability.

The existing evidence on dog ownership in older adulthood and brain and cognitive aging is mixed (Applebaum et al., 2022; Batty et al., 2017; Branson et al., 2016; Branson & Cron, 2022; Friedmann et al., 2020; Li et al., 2023; Taniguchi et al., 2023; Friedmann et al., 2023), likely due to key variations and limitations in study designs. For example, many studies examine the impact of pet ownership, (Applebaum et al., 2022; Branson & Cron, 2022; Li et al., 2023), not dog ownership specifically. Even fewer (Friedmann et al., 2023; Friedmann et al., 2020) consider dog walking behavior. Only one study has included a brain biomarker outcome, and the study was not focused on older adults (McDonough et al., 2022). The hypothesis-testing study that will result from the current pilot work will make a major contribution to this literature by including careful assessment of plausible mechanisms, moderator and mediator analyses, and both brain and cognitive outcomes.

A key lesson learned from this pilot work is that recruitment strategies must be modified to ensure a more representative sample and greater variability in mechanisms (*e.g.*, social connection, physical activity, dog walking). A multi-site study that reaches older adults in different geographic regions would be ideal. Less reliance on university-affiliated recruitment methods (*e.g.*, press release, newsletters, recruitment databases) and more emphasis on recruitment *via* social media and networking apps (*e.g.*, Facebook, Nextdoor) and in-person (at libraries, senior centers, and other community centers) may also help reach a more diverse group of older adults and improve external validity.

The hypothesis-testing study that results from this work should also use propensity score matching or a similar approach to match dog owners and non-dog owners on key sociodemographic factors. A major limitation of the broader literature on pets and health outcomes is the inability to fully account for differences between people who choose to (and can) own dogs and those who do not (or cannot) own dogs. It is possible that systematic differences in sociodemographic factors explain group differences in physical activity and health. For example, a study of adults ages 50-100 found pet owners were less likely to be Black or to live alone and more likely to be married/partnered, to work, and to live in single-family housing (Friedmann et al., 2023). In addition to matching on key sociodemographic variables (*e.g.*, age, socioeconomic status) that could confound the relationship between dog ownership and brain health, researchers should consider additional factors that may influence social connectedness or dog walking behavior (*e.g.*, personality traits, dog characteristics, neighborhood walkability) in their analyses.

Strengths of this pilot study include thorough assessment of physical activity using both device-based and self-reported methods, assessments of social connection using both general and dog-specific measures, and both cognitive and brain health (MRI) outcome measures. As discussed above, the sample lacked sociodemographic diversity (which will limit external validity in the larger study, if not addressed) and lacked variability in the key mechanisms of interest (which will limit the ability to test hypotheses in the larger study, if not addressed). The cross-sectional design is a limitation in that it cannot determine whether people become more active and social through dog ownership, or whether people who are more active and social are more likely to become dog owners.

## Conclusions

In conclusion, there are several compelling pathways by which dog ownership in older adulthood may support healthy brain and cognitive aging. This pilot work was a key first step in a line of research that aims to rigorously investigate this relationship. This study confirmed feasibility of data collection procedures while identifying important modifications needed for the success of the hypothesis-testing study, namely that recruitment strategies must be modified to ensure a more representative study sample with greater variability in mechanisms of interest (*e.g.*, social connection, physical activity, dog walking). The larger study should also make sure to match dog owners and non-dog owners on key sociodemographic factors that could confound the relationship between dog ownership and brain health. If this line of work ultimately demonstrates dog ownership in older adulthood supports healthy brain and cognitive aging (through our hypothesized mechanisms or otherwise), next steps would be to identify barriers to dog ownership and walking among older adults (*e.g.*, housing restrictions on pets, financial costs of pet ownership, fear of falling) and design interventions at various levels to address those barriers. Interventions that target older adults at higher risk for cognitive decline, such as those who live alone, could be especially impactful.

## Supplemental Information

10.7717/peerj.21560/supp-1 Supplemental Information 1 Dog ownership and brain health pilot study demographic, survey, physical activity, and cognition data

10.7717/peerj.21560/supp-2 Supplemental Information 2 Potter neuroimaging data

10.7717/peerj.21560/supp-3 Supplemental Information 3 Strobe checklist

## Additional Information and Declarations

### Data Availability

The following information was supplied regarding data availability: The raw data are available in the Supplemental Files.
